# Comparison of the feasibility and safety between distal transradial access and conventional transradial access in patients with acute chest pain: a single-center cohort study using propensity score matching

**DOI:** 10.1186/s12877-023-04058-y

**Published:** 2023-06-03

**Authors:** Wenhua Li, Juan Wang, Xiaofang Liang, Qiang Wang, Tao Chen, Yanbin Song, Ganwei Shi, Feng Li, Yong Li, Jianqiang Xiao, Gaojun Cai

**Affiliations:** 1 Department of Cardiology, Wujin Hospital Affiliated With Jiangsu University, the Wujin Clinical College of Xuzhou Medical University, No. 2 Yongning North Road, Tianning District, Changzhou City, Jiangsu Province 213002 China; 2 Department of Cardiothoracic Surgery, Wujin Hospital Affiliated With Jiangsu University, the Wujin Clinical College of Xuzhou Medical University, Jiangsu Province Changzhou City, China

**Keywords:** Acute chest pain, Emergency procedure, Radial artery occlusion, Distal transradial access

## Abstract

**Background:**

Distal transradial access (dTRA) has been suggested to have great advantages over cTRA. However, there is a lack of preliminary data on dTRA in patients undergoing emergency coronary angiography (CAG) or percutaneous coronary intervention (PCI). To explore the feasibility and safety of distal transradial access in patients with acute chest pain.

**Methods:**

A total of 1269 patients complaining of acute chest pain in our emergency department from January 2020 to February 2022 were retrospectively included. The patients who met the inclusion criteria were divided into the conventional transradial access (cTRA) group (*n* = 238) and the dTRA group (*n* = 158). Propensity score matching was used to minimize the baseline differences.

**Results:**

The cannulation success rate in the dTRA group was significantly lower than that in the cTRA group (87.41% vs. 94.81%, *p* < 0.05). No significant differences in the puncture time and total procedure time were noted between the two groups (*p* > 0.05). Compared with the cTRA group, the hemostasis duration was significantly shorter [4(4, 4) h vs. 10(8, 10) h, *p* < 0.001) and the incidence of minor bleeding (BARC Type I and II) was significantly lower in the dTRA group than that in the cTRA group (0.85% vs. 5.48%, *p* = 0.045). Asymptomatic radial artery occlusion was observed in six patients (5.83%) in the cTRA group and one patient (1.14%) in the dTRA group (*p* = 0.126). The subgroup analysis of ST-elevation myocardial infarction (STEMI) showed no significant differences in the puncture time, D-to-B time or total procedure time between the two groups.

**Conclusions:**

The dTRA for emergency CAG or PCI has an acceptable success rate and puncture time, a shorter hemostasis time, and a downward trend in RAO rate compared to the cTRA. The dTRA did not increase the D-to-B time in emergency coronary interventions in STEMI patients. On the contrary, a low incidence of RAO by the dTRA created an opportunity for future coronary interventions in non-culprit vessels in the same access.

**Trial registration:**

Retrospectively registered in Chinese Clinical Trial Registry (registry number: ChiCTR2200061104, date of registration: June 15, 2022).

**Supplementary Information:**

The online version contains supplementary material available at 10.1186/s12877-023-04058-y.

The diagnosis of acute chest pain is a challenge for attending physicians in the emergency department due to a wide spectrum of diseases associated with chest pain, including acute coronary syndrome (ACS), acute pulmonary embolism (APE), aortic dissection (AD), and harmless muscular tension [1]. In addition to a 12-channel electrocardiogram (ECG) and laboratory tests, cardiac imaging, including echocardiography and coronary angiography (CAG), plays a key role in the diagnosis. Conventional transradial access (cTRA) for CAG and percutaneous coronary intervention (PCI) has been widely accepted as the default vascular access, owing to less bleeding complications, early ambulation and lower all-cause mortality in patients with ACS [2, 3]. However, postprocedure radial artery occlusion (RAO) is a concern, with an incidence of approximately 5-8% [4], which precludes the use of the same artery for future transradial access (TRA) procedures, coronary artery bypass grafting surgery and hemodialysis arteriovenous fistula [5]. Recently, a novel puncture access site in the anatomical snuffbox (AS), namely, distal radial access (DRA), was first introduced by Kiemeneij for coronary catheterization in 2017 [6]. Some advantages of distal transradial access (dTRA) over cTRA were demonstrated, such as the lower incidence of RAO, shorter hemostasis duration, lower risk of bleeding complications and better comfort of the patient [7]. However, there is a lack of preliminary data on dTRA in patients with acute chest pain, who often require rapid diagnosis by CAG or treatment with potent anti-thrombotic agents or PCI. In this study, we aimed to investigate the real-world feasibility and safety of the dTRA in patients with acute chest pain who underwent emergency CAG or PCI using propensity score matching (PSM).

## Methods

We retrospectively analyzed a total of 1269 consecutive patients who complained of acute chest pain in our emergency department, which has a chest pain unit, from January 2020 to February 2022. We included the patients with acute chest pain who undergo emergency CAG or PCI for further study. The exclusion criteria were as follows: (1) patients undergoing CAG or PCI via femoral artery access without a first attempt on conventional radial access (CRA) or DRA; (3) age < 18 years; (4) patients without a palpable pulse in the conventional radial artery or the AS area; (5) infection of the puncture site; (6) a history of radial artery puncture and cannulation; and (7) patients with insufficient data. According to the puncture site, the enrolled patients were divided into the cTRA group and the dTRA group. This study involving human participants was carried out in accordance with the 1975 Helsinki Declaration, as revised in 2000 (5), and was approved by the Ethics Committee of Affiliated Wujin Hospital of Jiangsu University (Ethics approval number:201938). Informed consent was obtained from all enrolled patients in the study.

A thorough clinical evaluation of patients with acute chest pain was performed, including medical history, physical examination, 12-channel ECG, cardiac troponin I (cTnI), D-dimer, cardiac imaging and further focused diagnostics. Once the patient was diagnosed with ACS or was suspected to have myocardial ischemia, emergency CAG or PCI was initiated. The patient with confirmed ACS immediately received 300 mg of aspirin, 180 mg of ticagrelor or 300–600 mg of clopidogrel and 3000–5000 U of unfractionated heparin. The use of antiplatelet agents and unfractionated heparin in patients with suspected myocardial ischemia depended on the clinical decision of physicians.

The procedures were performed by 4 experienced radial operators who performed at least 300 CAG or PCI procedures via the cTRA per year and who had more than 5 months of training on the dTRA. The number of included cases in cTRA (dTRA) for each of the 4 operators was 60 (30), 42 (58), 42 (54) and 44 (66), respectively. The right radial artery was the primary access side. The choice of puncture site being at the proximal 3 cm of the wrist’s transverse striation for cTRA or at the proximal part of the AS or the first intermetacarpal space for dTRA was primarily based on the operator’s personal preference.

After disinfection, local anesthesia was achieved using approximately 2-3 ml of 2% lidocaine hydrochloride. The radial artery at the proximal part of the AS (an angle of 60-70°) or the first intermetacarpal space (an angle of 20–30°) or the wrist (an angle of 30-45°) was punctured using a 20G ago-cannula-needle. Following successful puncture, an angled tip of 0.025″ plastic guide wire (Terumo Corporation, Tokyo, Japan) was inserted into the cannula. A 0.014″ coronary guide wire was used as an alternative when resistance was encountered due to the excessive tortuosity of the radial artery. Then, a 6 French sheath (Terumo Corporation, Tokyo, Japan) was advanced into the artery through the plastic guide wire. The puncture time was recorded from the beginning of the first attempt to puncture to the moment of sheath insertion. Cannulation success was defined as completion of the sheath insertion in the same access. If unfractionated heparin was not administered in the emergency department, 3000 U of unfractionated heparin was injected through the sheath. A dose of 100 mcg of nitroglycerine was given to prevent radial spasm unless there was hemodynamic compromise. Then, emergency CAG was conducted. In the case of PCI, additional unfractionated heparin (70–100 U/kg) was administered according to the patient’s body weight. The total procedural time was defined as the duration from the beginning of local anesthesia to the end of CAG or PCI.

Following the emergency CAG or PCI procedure, hemostasis was immediately obtained using cohesive elastic bandage with a 4 × 4 cm piece of sterile gauze for the dTRA during the sheath removal. After 2–4 h, the bandage was loosened to check for hemostasis by nurses and was recompressed for additional 30-60 min in case bleeding until hemostasis. In the cTRA, hemostasis was achieved with an air-filled closure device. Briefly, when the sheath was pulled out, the compression device was placed over the puncture site at inflation of 8-12 ml of air into the air compartment without compromising the radial artery patency, which can be accessed by reverse Barbeau test. After 4 h, the deflation of 2 ml of air was performed per hour until the compression was completely released. Once bleeding, extra 2 ml of air was inflated into the air compartment for additional 60-90 min. The hemostasis duration was defined as the duration from the removal of the sheath to complete decompression. The bleeding complication was defined according to the BARC criteria [8]. Minor bleeding complications included BARC I type and II type. Access-related hematoma was assessed according to the Early Discharge After Transradial Stenting of Coronary Artery (EASY) trial classification [9]. The blood flow of the radial artery at the forearm and the AS was assessed by vascular ultrasonography (HS1 Plus, L18-4, Konica Minolta, Japan) during the follow-up period, which ranged from 2 to 24 months. RAO was determined by the absence of a color Doppler blood flow signal. The D-to-B time was expressed as the duration from hospital arrival to balloon inflation at the culprit lesion.

Demographics, medical history, procedure-related characteristics and follow-up data of the two groups were collected. The data were as follows: sex, age, medical history, the cause of acute chest pain, body mass index (BMI), mean artery pressure (MAP), heart rate (HR), hemoglobin (HB), platelet (PLT), creatinine (Cr), alanine aminotransferase (ALT), albumin (A), triglyceride (TG), total cholesterol (TC), high-density lipoprotein cholesterol (HDL-C), low-density lipoprotein cholesterol (LDL-C), puncture time, cannulation success rate, fluoroscopy time, total procedural time, contrast dosage, incidence of cardiogenic shock (CS), hemostasis duration, access-related complications including hematoma, bleeding, thumb numbness, arteriovenous fistula, pseudoaneurysm and RAO and the prognosis of disease. In addition, the causes of access failure were analyzed, including puncture failure and guide wire insertion failure.

Continuous variables were reported as the means ± standard deviations (SD) or median (interquartile ranges), as appropriate. For normal distribution, the differences between the two groups were compared using unpaired t tests. For nonnormal distributions, the differences between the two groups were analyzed with the Mann-Whitney U test. Categorical variables were expressed as counts and percentages, which were analyzed with Pearson’s chi-square test or Fisher's exact test.

To minimize biased estimates, the baseline differences and potential confounders were adjusted by 1:1 PSM using the nearest neighbor matching algorithm with a caliper of 0.1 and without replacement. The covariates in the PSM model included sex, age, diabetes mellitus (DM), smoking, hypertension, ACS, BMI, MAP, HR, HB, PLT, Cr, ALT, HDL-C, LDL-C, transient hypotension, CS and use of anticoagulation preprocedure. Comparison of the standardized mean difference was used to evaluate the match quality. Standardized differences less than 10% indicated a relatively good match quality. All data were analyzed using SPSS version 22.0 (IBM Co., Armonk, NY, USA). A *p* value < 0.05 was considered statistically significant.

## Results

**Fig. 1:**
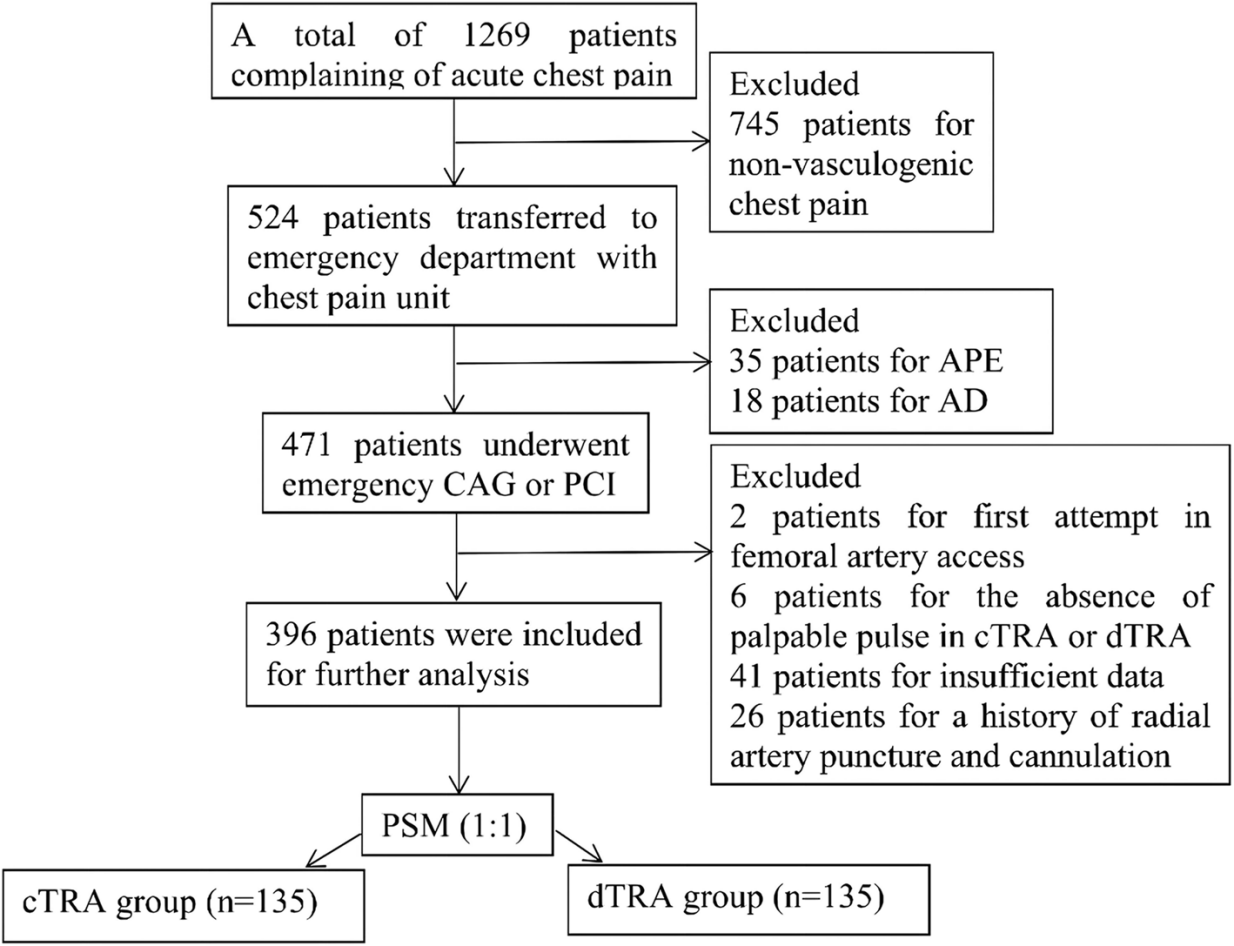
Study flowchart. APE, acute pulmonary embolism; AD, aortic dissection; CAG, coronary angiography; PCI, percutaneous coronary intervention; cTRA, conventional transradial access; dTRA, distal transradial access

A total of 1269 patients complaining of acute chest pain visited our emergency department from January 2020 to February 2022. Patients were excluded based on the inclusion and exclusion criteria. Therefore, a total of 396 patients (*n* = 238 for the cTRA group; *n* = 158 for the dTRA group) were included for further analysis. Pairs of patients in the two groups were successfully matched by 1:1 PSM. (Fig. 1).

**Fig. 2:**
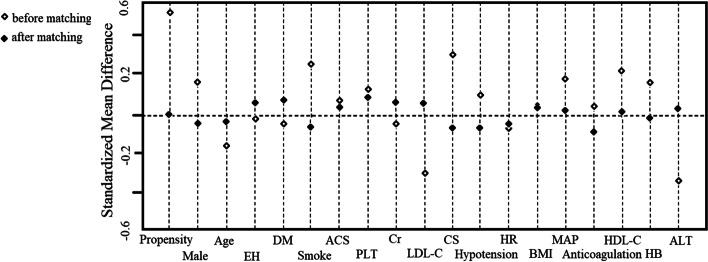
The clinical baseline data of 18 confounding variables before and after matching between the two groups. After matching, the standardized mean difference was less than 10% between the two matched groups. EH, essential hypertension; DM, diabetes mellitus; ACS, acute coronary syndrome; PLT, platelet; Cr, creatinine; LDL-C, low-density lipoprotein cholesterol; CS, cardiogenic shock; HR, heart rate; BMI, body mass index; MAP, mean artery pressure; HDL-C, high-density lipoprotein cholesterol; HB, hemoglobin; ALT, alanine aminotransferase

Some of the clinical baseline data of the enrolled patients before and after matching are summarized in Table 1. There were significant differences in the percentages of smoking and CS and the levels of LDL-C and HDL-C between the two groups before matching. In the study, a standardized mean difference less than 10% revealed high-quality matching and an adequate balance of covariate distribution between the two groups (Fig. 2). There were no significant differences in 18 confounding variables between the two groups after matching (Fig. 2, Table 1).

**Table 1:** Comparison of the clinical baseline data

characteristics	Before matching		*χ*2 (*Z*) (t)	*p*	After matching		χ2 (Z) (t)	*p*
	**cTRA (*n* = 238)**	**dTRA (*n* = 158)**			**cTRA (*n* = 135)**	**dTRA (*n* = 135)**		
Age [M (P_25_, P_75_)] (years)	67.00 (55.00, 74.25)	65.00 (52.00, 74.00)	-1.675	0.094	65.00 (54.00, 72.00)	64.00 (52.00, 73.00)	-0.642	0.521
Male [n (%)]	170 (71.43%)	125 (79.11%)	2.952	0.086	107 (79.26%)	103 (76.30%)	0.343	0.558
BMI [M (P_25_, P_75_)] (kg/m2)	24.51 (22.15, 26.48)	24.22 (21.97, 27.10)	-0.023	0.982	24.77 (22.49, 26.42)	24.22 (22.04, 27.31)	-0.180	0.857
EH [n (%)]	164 (68.91%)	110 (69.62%)	0.023	0.880	99 (73.33%)	95 (70.37%)	0.293	0.588
DM [n (%)]	75 (31.51%)	47 (29.75%)	0.139	0.709	40 (29.63%)	45 (33.33%)	0.429	0.512
Smoke [n (%)]	116 (48.74%)	96 (60.76%)	5.515	0.019*	84 (62.22%)	78 (57.78%)	0.556	0.456
HB [M (P_25_, P_75_)] (g/L)	141.00 (131.00, 153.00)	147.50 (132.00, 155.00)	-1.772	0.076	145.00 (131.00, 157.00)	148.00 (134.00, 155.00)	-0.223	0.824
PLT [M (P_25_, P_75_)] (*10^9^/L)	204.50 (171.75, 239.25)	211.50 (180.00, 252.25)	-1.188	0.235	211.00 (174.00, 247.00)	212.00 (181.00, 249.00)	-0.480	0.631
Cr [M (P_25_, P_75_)] (umol/L)	74.00 (63.00, 91.00)	74.00 (64.00, 87.25)	-0.591	0.555	73.00 (63.00, 88.00)	73.50 (63.00, 87.00)	-0.304	0.761
ALT [M (P_25_, P_75_)] (u/L)	33.00 (20.00, 58.00)	31.00 (21.00, 58.00)	-0.913	0.361	32.00 (19.00, 55.00)	31.00 (21.00, 58.00)	-0.281	0.778
HDL-C [M (P_25_, P_75_)] (mmol/L)	1.12 (0.97, 1.32)	1.21 (0.99, 1.49)	-2.458	0.014*	1.14 (0.98, 1.38)	1.16 (0.99, 1.43)	-0.574	0.566
LDL-C [M (P_25_, P_75_)] (mmol/L)	2.95 (2.33, 3.45)	2.74 (2.26, 3.27)	-2.419	0.016*	2.81 (2.21, 3.31)	2.87 (2.35, 3.29)	-0.566	0.572
HR [M (P_25_, P_75_)] (bpm)	79.50 (70.00, 92.25)	78.00 (70.00, 90.00)	-0.569	0.569	79.00 (69.00, 93.00)	78.00 (69.00, 90.00)	-0.656	0.512
MAP [M (P_25_, P_75_)] (mmHg)	94.33 (84.58, 107.08)	96.83 (87.67, 104.67)	-1.426	0.154	97.68 ± 16.38	98.13 ± 14.47	-0.236	0.814
ACS [n (%)]	225 (87.21%)	146 (92.41%)	0.730	0.393	128 (94.81%)	127 (94.07%)	0.071	0.790
CS [n (%)]	25 (10.50%)	7 (4.43%)	4.716	0.030*	5 (3.70%)	7 (5.19%)	0.349	0.555
Transient hypotension [n (%)]	80 (33.61%)	47 (29.75%)	0.652	0.420	38 (28.15%)	42 (31.11%)	0.284	0.594
Anticoagulation preprocedure [n (%)]	217 (91.18%)	141 (89.24%)	0.410	0.522	122 (90.37%)	126 (93.33%)	0.792	0.374

*cTRA* conventional transradial access, *dTRA* distal transradial access, *BMI* body mass index, *EH* essential hypertension, *DM* diabetes mellitus, *HB* hemoglobin, *PLT* platelet, *Cr* creatinine, *ALT* alanine aminotransferase, *A* albumin, *TG* triglyceride, *TC* total cholesterol, *HDL-C* high-density lipoprotein cholesterol, *LDL-C* low-density lipoprotein cholesterol, *HR* heart rate, *MAP* mean artery pressure, *ACS* acute coronary syndrome, *CS* cardiogenic shock

The before and after matching values are presented as the mean ± SD, median (interquartile range), or number (%). A *p* value < 0.05 was considered statistically significant. *p values < *0.05*

The time from the onset of acute chest pain to visiting the emergency department in the dTRA group was significantly longer than that in the cTRA group [5 (3, 10) h vs. 4 (2, 6) h, *p* < 0.01]. The cannulation success rate in the dTRA group was significantly lower than that in the cTRA group (87.41% vs. 94.81%, *p* < 0.05). Interestingly, no significant differences in the puncture time and total procedure time were noted between the two groups [2 (1, 3) min vs. 2 (1, 3) min, *p* = 0.625; 45 (30, 60) min vs. 40 (30, 55) min, *p* = 0.276, respectively). There were no significant differences in the procedure methods, percentage of ACS, number of stents, contrast dosage or 90-day mortality between the two groups (*p* > 0.05). Moreover, no significant differences were observed in constituent ratios of ACS [ST-elevation myocardial infarction (STEMI), non-STEMI (NSTEMI) and unstable angina (UA)] and culprit vessel [left main artery (LM), left anterior descending artery (LAD), left circumflex artery (LCX) and right coronary artery (RCA)] between the two groups (*p* > 0.05) (Table 2).

**Table 2:** Comparison of the emergency procedure characteristics between the matched groups

	cTRA group (*n* = 135)	dTRA group (*n* = 135)	χ2 (Z) (t)	*P*
Time from onset to visit [M (P_25_, P_75_)] (h)	4 (2, 6)	5 (3, 10)	-3.032	0.002**
Puncture time [M (P_25_, P_75_)] (min)	2 (1, 3)	2 (1, 3)	-0.451	0.652
Cannulation success rate [n (%)]	128 (94.81%)	118 (87.41)	4.573	0.032*
Total procedure time [M (P_25_, P_75_)] (min)	40 (30, 55)	45 (30, 60)	-1.089	0.276
PCI [n (%)]	119 ( 88.15%)	107 (79.26%)	3.910	0.048*
ACS [n (%)]	128 (94.81%)	127 (92.03%)	0.071	0.790
STEMI [n (%)]	90 (66.67%)	84 (62.22%)	0.582	0.446
NSTEMI [n (%)]	37 (27.41%)	37 (27.41%)	0.000	1.000
UA [n(%)]	1 (0.74%)	6 (4.44%)	3.666	0.056
Culprit vessel				
LM [n (%)]	1 (0.74%)	2 (1.48%)	0.337	0.562
LAD [n (%)]	70 (51.85%)	58 (42.96%)	2.139	0.144
LCX [n (%)]	17 (12.59)	19 (14.07%)	0.128	0.720
RCA [n (%)]	39 (28.89)	43 (31.85%)	0.280	0.597
Number of stents[M (P_25_, P_75_)]	1 (1, 1)	1 (0, 2)	-0.080	0.936
Contrast dosage[M (P_25_, P_75_)]	120 (100, 150)	110 (70, 130)	-1.351	0.177
Cardiac mortality [n (%)]	3 (2.22%)	5 (3.70%)	-	0.722

*cTRA* conventional transradial access, *dTRA* distal transradial access, *PCI* percutaneous coronary intervention, *ACS* acute coronary syndrome, *STEMI* ST-elevation myocardial infarction, *NSTEMI* non-ST-elevation myocardial infarction, *UA* unstable angina, *LM* left main artery, *LAD* left anterior descending artery, *LCX* left circumflex artery, *RCA* right coronary artery

Values are presented as the mean ± SD, median (interquartile range), or number (%). A *p* value < 0.05 was considered statistically significant. *p values < 0.05, **p values < 0.01

**Fig. 3:**
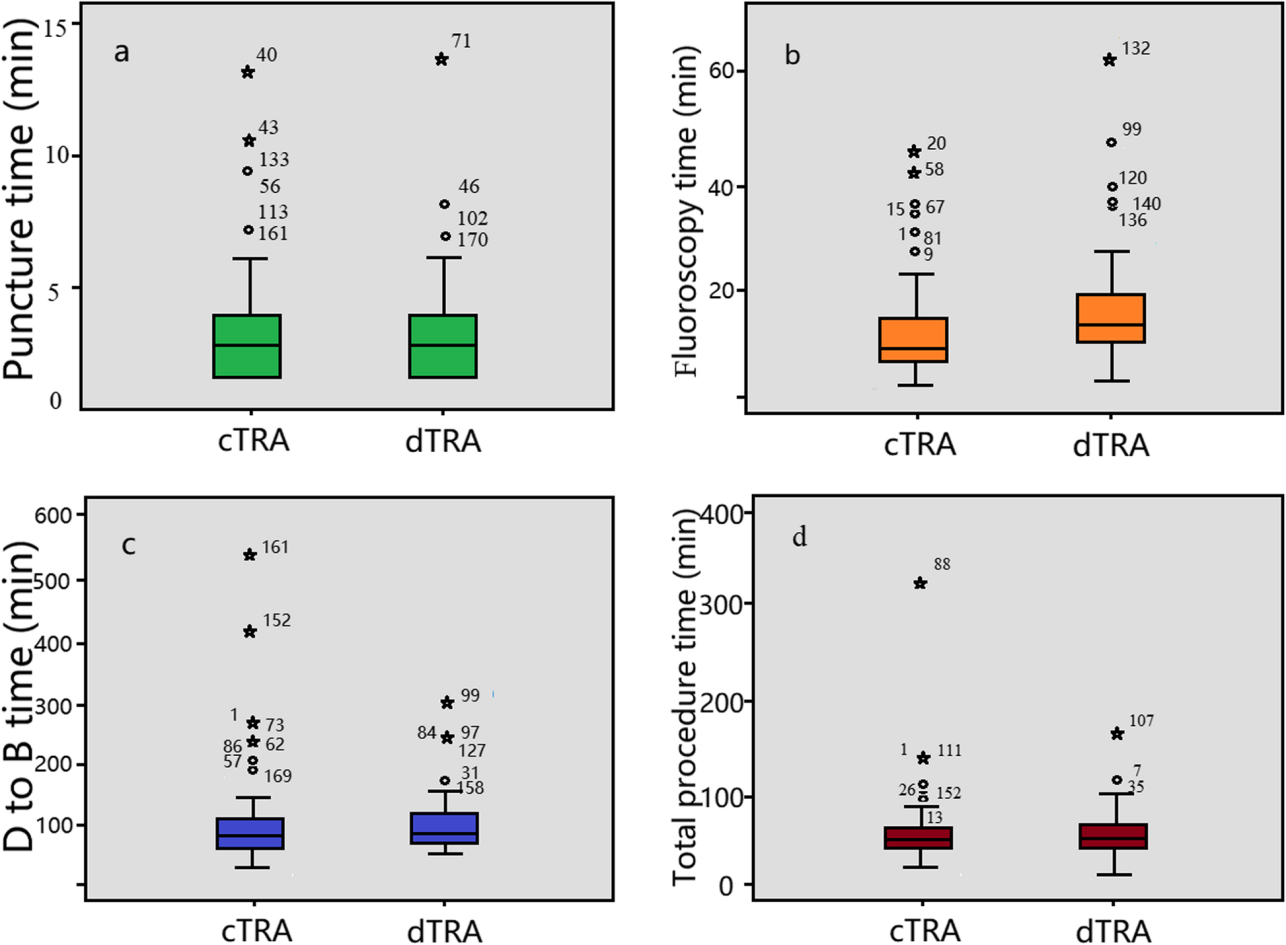
Comparison of time associated with emergency procedures in patients with ST-elevation myocardial infarction between the matched groups. No significant differences were noted in puncture time (**a**), fluoroscopy time (**b**), D to B time (**c**) and total procedure time (**d**) between the matched subgroups. D to B, door-to-balloon; cTRA, conventional transradial access; dTRA, distal transradial access

The difference in the percentage of STEMI was not significant between the cTRA group and the dTRA group [90 (66.67%) vs. 84 (62.22%), *p* > 0.05]. No significant difference in the cannulation success rate was reported between the two groups [86 (95.56%) vs. 76 (90.48%), *p* = 0.186]. The subgroup analysis based on the presence of STEMI showed that there were no significant differences in puncture time, D-to-B time or total procedure time between the two groups (*p* > 0.05). However, the fluoroscopy time in the dTRA group was significantly longer than that in the cTRA group [11.53 (7.43, 17.54) min vs. 9.13 (7.24, 14.12) min, *p* = 0.039] (Fig. 3).

**Fig. 4:**
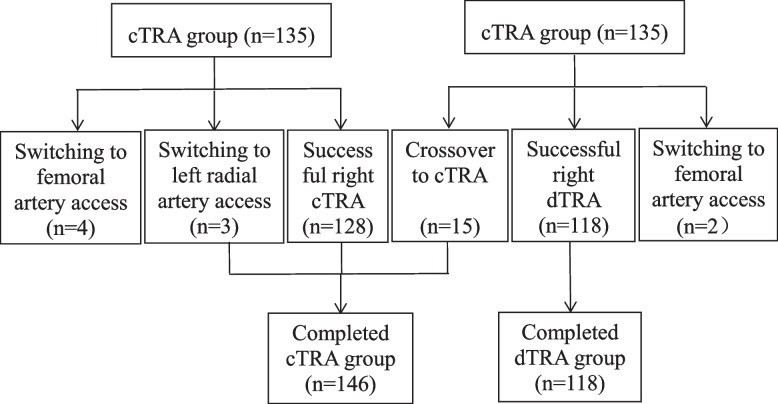
The crossover and switched access between the two groups. cTRA, conventional transradial access; dTRA, distal transradial access

Switched or crossover access was observed in 7 out of 135 patients (5.19%) in the cTRA group and in 17 (12.59%) in the dTRA group (*p* = 0.032). The cTRA was actually completed in 146 patients and the dTRA in 118 patient (Fig. 4). There were no significant differences in baseline data between the two groups after crossover (supplementary Table). The major causes of puncture procedure failure, including puncture failure and guide wire insertion failure, are listed in Table 3. In the dTRA group, resistance was encountered in 16 cases of tortuosity of the radial artery during wire guide insertion. Of these, sheath introduction was achieved via the 0.014″ Runthrough NS guide wire (Terumo Corporation, Tokyo, Japan) in 8 cases.

**Table 3:** Comparison of the failure causes between the two actual completed groups

	Unsuccessful cTRA (*n* = 7)	Unsuccessful dTRA (*n* = 17)	*P*
Puncture failure [n (%)]	6 (85.71%)	9 (52.94%)	0.191
Guide wire insertion failure [n (%)]	1 (14.29%)	8 (47.06%)	0.191

*cTRA* conventional transradial access, *dTRA* distal transradial access

A *p* value < 0.05 was considered statistically significant

The hemostasis duration in the cTRA group was significantly longer than that in the dTRA group [10 (8, 10) h vs. 4 (4, 4) h, *p* < 0.001]. The incidence of minor bleeding (BARC type I and II) was significantly higher in the cTRA group than in the dTRA group (5.48% vs. 0.85%, *p* = 0.045). No significant difference was noted in hematoma or numbness (*p* > 0.05). Local numbness in the puncture site was experienced in 2 (1.37%) patients in the cTRA group and 1 (0.85%) in the dTRA group, which spontaneously improved within 2 weeks. No pseudoaneurysms or arteriovenous fistulas were observed in our study (Table 4).

**Table 4:** Comparison of safety between the two groups

	cTRA group (*n* = 146)	dTRA group (*n* = 118)	**χ2** (Z)	*P*
Hemostasis duration [*M* (P25, P75), h]	10 (8, 10)	4 (4, 4)	-12.228	< 0.001***
Bleeding [n (%)]	8 (5.48%)	1 (0.85%)	-	0.045*
Hematoma [n (%)]	4 (2.74%)	0 (0%)	-	0.383
Numbness [n (%)]	2 (1.37%)	1 (0.85%)	-	1.000
	cTRA group (*n* = 112)	dTRA group (*n* = 86)		
Pseudoaneurysm [n (%)]	0 (0%)	0 (0%)	-	1.000
Arteriovenous fistula [n (%)]	0 (0%)	0 (0%)	-	1.000
Follow-up rate [n (%)]	*n* = 103 (70.55%)	*n* = 88 (74.58%)	0.529	0.467
Follow-up period [M (P_25_, P_75_), month]	12 (7, 17)	11 (5, 15)	-1.462	0.144
RAO [n (%)]	6 (5.83%)	1 (1.14%)	-	0.126

*cTRA* conventional transradial access, *dTRA* distal transradial access, *RAO* radial artery occlusion

Values are presented as the median (interquartile range) or number (%). A *p* value < 0.05 was considered statistically significant. *p values < *0.05,* ***p values < *0.001*

**Fig. 5:**
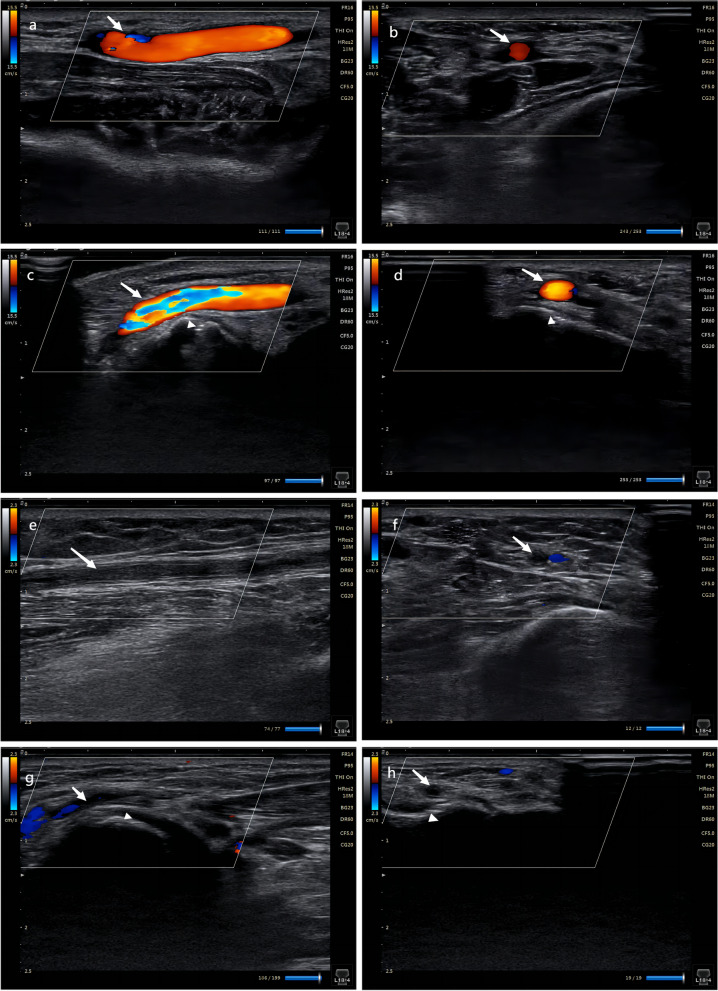
Color Doppler images of the proximal RA and the distal RA. Long axis (**a**) and short axis (**b**) images of a normal proximal RA (white arrow). Long axis (**c**) and short axis (**d**) images of a normal distal RA (white arrow). Long axis (**e**) and short axis (**f**) images of an occluded proximal RA (white arrow). Long axis (**g**) and short axis (**h**) images of an occluded distal RA (white arrow). The distal RA lies on the dorsal surface of the scaphoid (arrowhead). RA, radial artery

A total of 103 of 146 patients (70.54%) in the cTRA group and 88 of 118 patients (74.58%) in the dTRA group were followed up by vascular ultrasonography during the follow-up period (*p* = 0.467). No significant difference was noted in the average follow-up period between the two groups [12 (7, 17) months vs. 11 (5, 15) months, *p* = 0.144). Asymptomatic proximal RAO was observed in 6 patients (5.83%) in the cTRA group and distal RAO in 1 patients (1.14%) in the dTRA group (*p* = 0.126) during the follow-up period (Fig. 5).

The possible factors associated with puncture complications and the hemostasis duration included CS, hypotension, use of anticoagulation preprocedure and postprocedure, unfractionated heparin dosage and the types of antiplatelet agents. No significant difference was noted in CS, hypotension or use of anticoagulation preprocedure between the two groups after matching (Table 1). Similarly, no significant difference was found in the unfractionated heparin dosage, the types of antiplatelet agents and the use of anticoagulation postprocedure (*p* > 0.05, Table 5).

**Table 5:** Comparison of the factors associated with puncture complications and hemostasis duration between the two groups

	cTRA group (*n* = 146)	dTRA group (*n* = 118)	χ2 (Z) (t)	*P*
Unfractionated heparin dosage [*M* (P25, P75), U]	8000 (7000, 8000)	8000 (7000, 8000)	-0.321	0.749
Antiplatelet agents				
Clopidogrel [n (%)]	9 (6.16%)	11 (9.32%)	0.929	0.335
Ticagrelor [n (%)]	131 (89.73%)	98 (83.05%)	2.528	0.112
Anticoagulation postprocedure [n (%)]	107 (73.29%)	82 (69.49%)	0.462	0.497

Values are presented as the mean ± SD, median (interquartile range), or number (%)

*cTRA* conventional transradial access, *dTRA* distal transradial access

A *p* value < 0.05 was considered statistically significant

## Discussion

In this retrospective observational study, the principal findings are as follows: i) the success rate of the emergency CAG or PCI via the dTRA was high and acceptable, despite some conversion of the access site; ii) the hemostasis duration in the dTRA group was significantly shorter than that in the cTRA group; iii) the dTRA did not delay the D-to-B time and total procedure time in patients with STEMI. To our knowledge, this was the first study to investigate the feasibility and safety of the dTRA for emergency CAG or PCI in patients complaining of acute chest pain.

Time is life for patients with acute chest pain, especially STEMI. A large-scale, multicenter, randomized trial suggested that cTRA in patients with ACS was associated with better clinical outcomes than transfemoral access (TFA) [10]. Therefore, the cTRA was recommended over the TFA in patients with STEMI by the 2017 ESC guidelines [11]. Recently, a novel puncture site, AS, has attracted more attention due to less bleeding, a short hemostasis duration and a low incidence of RAO [12]. However, a review demonstrated that most procedural indications (96.2%) avoided the dTRA in an emergency context due to concerns about possible clinical adverse events [13]. Only a few studies showed no significant difference in adverse cardiac events in the dTRA and cTRA [13, 14]. In our study, no significant difference was observed in cardiac mortality between the two groups (2.22% vs. 3.70%, *p* > 0.05) despite a longer time from onset to visit [4 (2, 6) h vs. 5 (3, 10) h, *p* = 0.002] and an increased fluoroscopy time in the subgroup analysis in STEMI due to more use of 0.014″ working guide wire in the dTRA group [11.53 (7.43, 17.54) min vs. 9.13 (7.24, 14.12) min, *p* = 0.039], which preliminarily indicated the effectiveness and feasibility of the dTRA in emergency CAG or PCI.

The cannulation success rate is an important index on which most studies have focused. Previous studies have reported a dTRA cannulation success rate of 70% to 99.2%, most of which were lower than that of the cTRA [12, 15]. The cannulation success rate of the dTRA was 89% in the pilot study of Kimeneij. In another study regarding STEMI, the success rate of the dTRA was 92.8% [16]. A recent meta-analysis showed that the cannulation success rate in dTRA was not significantly different compared with cTRA (*p* = 0.1) [17]. However, in our trial, the cannulation success rate of the dTRA was significantly lower than that of the cTRA (87.41% vs. 94.81%, *p* = 0.032). Meanwhile, the 87.41% cannulation success rate of the dTRA in the present study was low compared to that in previous studies. The possible explanations are as follows: 1) patients with acute chest pain were included in our study, and all of them underwent emergency CAG or PCI. In the emergency context, acute pain and the first unsuccessful puncture can induce more spasms of the small radial artery in the AS, resulting in puncture failure and requiring the puncture access technique to be changed; 2) anticoagulation agents were used in 93.33% patients before the procedure due to a high percentage of ACS (92.03%) in the present trial, which made the puncture more difficult in case of the first unsuccessful puncture resulting in possible subcutaneous hematomas; 3) a high percentage of STEMI (62.22%) patients were included in the study. The principle that time is heart muscle was followed in the setting of STEMI. The puncture access was immediately switched to avoid undue delay of D-to-B time after no more than three unsuccessful attempts, which can be proven by the fact that there was no significant difference in the puncture time [2 (1, 3) min vs. 2 (1, 3) min, *p* > 0.05] and D-to-B time [65 (65, 75) min vs. 65 (40,80) min, *p* > 0.05] between the two groups. 4) Tortuosity of the radial artery in the AS was observed in 16 patients in the dTRA group and in 1 patient in the cTRA group, which resulted in cannulation failure in 8 patients in the dTRA group and in 1 patient in the cTRA group, despite the assistance of a 0.014″ working guide wire. 5) The factors affecting the success rate of punctures are mainly associated with the personal experience and proficiency of the operator. In our study, 5 months of training on the dTRA was still relatively short. Considering the abovementioned, the puncture success rate of the dTRA (87.41%) in our study may be acceptable.

In the emergency context, the dTRA puncture time is a concern as it may delay the D-to-B time. A review about dTRA in STEMI indicated that the puncture time of the dTRA did not affect the timely opening of the culprit vessel [18]. Kim Y reported that the mean snuffbox puncture time was 2.7 ± 1.6 min in patients with STEMI, which did not delay the D-to-B time [16]. YJ Wang showed that puncture time in the dTRA group was longer than that in the cTRA group [2.4 (1.7–4.2) min vs. 1.7 (1.4–2.3) min; *p* < 0.001) whereas the door-to-wire time was not delayed in patients with STEMI [71 (54–88) min vs. 64 (56–82) min, *p* = 0.103] [19]. Similarly, our study showed that the dTRA puncture time was 2 (1, 3) min, and no significant differences were observed in D-to-B time [65 (65, 75) min vs. 65 (40, 80) min, *p* > 0.05] between the dTRA group and the cTRA group. Echo-guided puncture of dTRA may serve to reduce the time to successful vascular access. However, it is not clear whether ultrasound preparation and the radial artery exploration increase the D-to-B time in the emergency CAG or PCI, and further studies are needed. Thus, the dTRA puncture time seemed to be acceptable in the present study. Given the puncture success rate, the puncture time and the D-to-B time, the dTRA might be feasible as an alternative access in patients with acute chest pain.

Previous studies have reported that a shorter hemostasis duration was found in dTRA patients than in cTRA patients [20, 21]. Another study found that 2 h hemostasis may be sufficient in patients undergoing CAG by a 5-F sheath [22]. Ji Woong Roh et al. reported that 3 h hemostasis for PCI using the dTRA was feasible [12]. In this study, the hemostasis duration in the dTRA group was significantly shorter than that in the cTRA group [4 (4, 4) vs. 10 (8, 10), *p* < 0.001]. The short hemostasis duration greatly improved patient satisfaction and reduced the nursing workload postprocedure. However, the median hemostasis duration in our study was 4 h, longer than that of Ji Woong Roh et al. due to the use of potent dual antiplatelet agents and a large dose of heparin in the emergency context. A shorter hemostasis time (2-3 h) will be tried in our future practice in order to explore the major advantages of the distal radial approach in emergency intervention with a high bleeding risk. Most previous studies showed that fewer complications in the dTRA, such as hematoma, hemorrhage, pseudoaneurysm and arteriovenous fistula, were observed compared with those in the cTRA [21, 23]. Similar to previous studies, the incidence of minor bleeding in the dTRA group was significantly lower than that in the cTRA group in our study (0.85% vs. 5.48%, *p* = 0.045). Further analysis of the factors associated with puncture complications showed that the difference was not significant in the types and dosage of antithrombotic agents during the peri-procedure period. Moreover, despite the potent antithrombotic treatment, minor bleeding was found in only one patient with emergency CAG or PCI in the dTRA group, and no hematomas developed in the patients in the dTRA group, which affirmed the safety of dTRA in emergency intervention with a high bleeding risk.

RAO is the most common complication of conventional transradial interventional therapy. The proportion of multivessel lesions was high in patients undergoing PCI (34.67%) [21], but the proportion was higher in patients with ACS (58.6%) [16]. Emergency PCI was performed first in the culprit vessel to save the ischemic myocardium. Then, nonculprit vessels were then considered for the scheduled PCI via the same access. Thus, it is particularly important to minimize the RAO incidence in the emergency context. A prospective clinical trial performed by Andrea Pacchioni et al. reported that the RAO incidence in DRA was 0.5%, which was much lower than that in CRA (0.5% vs. 4.8%, *p* < 0.01) [24]. A dramatic reduction in RAO after dTRA was observed in the study of Eid-Lidt et al. [25] and YJ Wang et al. [19]. A recent meta- analysis showed that dTRA was associated with a significant lower risk of RAO (risk ratio [RR]: 0.36; 95% CI:0.23-0.56; *p* < 0.001) in comparison to cTRA [26]. In our study, a 1:1 PSM was performed to adjust for the potential confounders that might lead to increased RAO, including CS, MAP, and transient hypotension. The difference was not significant in these confounders after matching (*p* > 0.05). However, a downward trend in asymptomatic RAO was still detected by ultrasonography in the dTRA group compared with the cTRA group [1/88 (1.14%) vs. 6/103 (5.83%), *p* = 0.126]. The non-significant difference in RAO between the two groups may be related to the relatively low follow-up rate of ultrasonography and small sample size. The reason for the lower RAO incidence in dTRA was unclear. The puncture site of DRA is beyond the rise of several anastomotic branches, which may allow avoiding flow interruption in the forearm radial artery and reducing the risk of proximal extension thrombosis and forearm RAO [27]. Therefore, the dTRA showed a great advantage in reducing the RAO incidence in the emergency context, which makes it possible to reuse the same access in hemodialysis patients and to be able to schedule coronary intervention in patients and candidates for coronary bypass.

Certainly, there were some limitations in this study. First, this was a single-center study with a small sample size. The lack of a multicenter, large-sample, randomized design cannot completely exclude the influence of potential confounders even if PSM was performed to adjust for the between-group differences in the baseline data. Second, only patients with well-palpable DRA were considered suitable for emergency CAG or PCI via dTRA in this study, which might lead to selection bias. Third, the time range of follow-up, from 2 months to 2 years, was very wide in different individuals. Therefore, the incidence of RAO may have been overestimated by neglecting the reversibility of RAO after 1-3 months [28]. Fourth, the follow-up rate in vascular ultrasonography was low in our study (70.54% in the cTRA group and 74.58% in the dTRA group) due to the COVID-19 outbreak, which might have influenced the results of our study. Fifth, the lack of a preprocedural vascular ultrasound evaluation is an inherent weakness of this retrospective study. Vascular ultrasound is an effective tool to evaluate the feasibility and safety of dTRA. However, preprocedural ultrasound evaluation of the anatomy and caliber of the radial artery requires several minutes, which might lead to delays in D-to-B time. Thus, it remains controversial in the emergency context.

## Conclusions

The dTRA for emergency CAG or PCI was feasible in terms of the cannulation success rate, puncture time and total procedure time in our study. The dTRA for emergency PCI did not increase D-to-B time in STEMI patients. Moreover, the dTRA showed a shorter hemostasis duration and a lower incidence of bleeding, hematoma and RAO in patients with acute chest pain, indicating that this puncture approach is safe in emergency CAG or PCI. A low incidence of RAO in emergency coronary interventions by the dTRA created an opportunity for future coronary interventions in non-culprit vessels in the same access.

## Supplementary Information

**Additional file 1:** **Supplementary Table. **Comparisons of the clinical baseline databetween the two groups after crossover.

## Data Availability

All authors had full access to the study data (including statistical reports and tables) and can take responsibility for the data integrity and the accuracy of the analysis. The datasets used and/or analysed during the current study are available from the corresponding author on reasonable request.

## Abbreviations

ACS: Acute coronary syndrome
APE: Acute pulmonary embolism
AD: Aortic dissection
ECG: Electrocardiogram
CAG: Coronary angiography
cTRA: Conventional transradial access
PCI: Percutaneous coronary intervention
RAO: Radial artery occlusion
TRA: Transradial access
AS: Anatomical snuffbox
DRA: Distal radial access
PSM: Propensity score matching
cTnI: Cardiac troponin I
BMI: Body mass index
MAP: Mean artery pressure
HR: Heart rate
HB: Hemoglobin
PLT: Platelet
Cr: Creatinine
ALT: Alanine aminotransferase
A: Albumin
TG: Triglyceride
TC: Total cholesterol
HDL-C: High-density lipoprotein cholesterol
LDL-C: Low-density lipoprotein cholesterol
CS: Cardiogenic shock
SD: Standard deviations
DM: Diabetes mellitus
STEMI: ST-elevation myocardial infarction
NSTEMI: Non ST-elevation myocardial infarction
UA: Unstable angina
LAD: Left anterior descending artery
LCX: Left circumflex artery
RCA: Right coronary artery
TFA: Transfemoral access
