# Supplementary material for: Comparison of the feasibility and safety between distal transradial access and conventional transradial access in patients with acute chest pain: a single-center cohort study using propensity score matching
Source: BMC Geriatr. 2023 Jun 3;23:348. doi: 10.1186/s12877-023-04058-y (PMC10238772; doi:10.1186/s12877-023-04058-y)
Supplement: Supplementary file 1 — Additional file 1: Supplementary Table. Comparisons of the clinical baseline databetween the two groups after crossover. [file 12877_2023_4058_MOESM1_ESM.doc]

**Supplementary table** Comparisons of the clinical baseline data between the two groups after crossover

| **characteristics** | **cTRA (n=146)** | **dTRA (n=1118)** | **χ2 (Z) (t)** | ***p*** |
| --- | --- | --- | --- | --- |
| Age [M (P_25_, P_75_)] (years) | 65.00 (54.00, 72.00) | 63.00 (52.00, 73.00) | -0.744 | 0.457 |
| Male [n (%)] | 116 (79.45%) | 91 (77.12%) | 0.21 | 0.655 |
| BMI [M (P_25_, P_75_)] (kg/m2) | 24.71 (22.49, 26.57) | 24.33 (22.06, 27.43) | -0.135 | 0.893 |
| EH [n (%)] | 107 (73.29%) | 82 (69.49%) | 0462 | 0.583 |
| DM [n (%)] | 43 (29.45%) | 41 (34.75%) | 0.843 | 0.425 |
| Smoke [n (%)] | 91 (62.32%) | 68 (57.63%) | 0.602 | 0.451 |
| HB [M (P_25_, P_75_)] (g/L) | 145.50 (132.00, 156.25) | 148.50 (134.00, 156.00) | -0.366 | 0.715 |
| PLT [M (P_25_, P_75_)] (*10^9^/L) | 212.50 (175.75, 247.25) | 205.50 (180.00, 246.00) | -0.053 | 0.958 |
| Cr [M (P_25_, P_75_)] (umol/L) | 72.00 (63.00, 88.00) | 75.00 (62.00, 87.00) | -0.115 | 0.908 |
| ALT [M (P_25_, P_75_)] (u/L) | 29.50 (19.00, 51.50) | 31.00 (22.00, 57.25) | -1.012 | 0.271 |
| HDL-C [M (P_25_, P_75_)] (mmol/L) | 1.16 (0.99, 1.39) | 1.16 (0.97, 1.42) | -0.054 | 0.957 |
| LDL-C [M (P_25_, P_75_)] (mmol/L) | 2.82 (2.22, 3.27) | 2.85 (2.30, 3.28) | -0.131 | 0.896 |
| HR [M (P_25_, P_75_)] (bpm) | 79.00 (71.00, 89.25) | 78.00 (68.00, 91.00) | -0.447 | 0.655 |
| MAP [M (P_25_, P_75_)] (mmHg) | 98.18 ± 14.57 | 97.94 ± 15.17 | 0.131 | 0.243 |
| ACS [n (%)] | 137 (93.84%) | 112 (94.92%) | 0.142 | 0.793 |
| CS [n (%)] | 4 (2.74%) | 6 (5.08%) | 0.985 | 0.350 |
| Transient hypotension [n (%)] | 37 (25.34%) | 38 (32.20%) | 1.510 | 0.272 |
| Anticoagulation preprocedure [n (%)] | 133 (91.09%) | 109 (92.37%) | 0.139 | 0.824 |

cTRA, conventional transradial access; dTRA, distal transradial access; BMI, body mass index; EH, [essential](javascript:;) [hypertension](javascript:;); DM, diabetes mellitus; HB, hemoglobin; PLT, platelet; Cr, creatinine; ALT, alanine aminotransferase; A, albumin; TG, triglyceride; TC, total cholesterol; HDL-C, high-density lipoprotein cholesterol; LDL-C, low-density lipoprotein cholesterol; HR, heart rate; MAP, mean artery pressure; ACS, acute coronary syndrome; CS, cardiogenic shock.
